# Supplementary material for: Visualization of very large high-dimensional data sets as minimum spanning trees
Source: J Cheminform. 2020 Feb 12;12:12. doi: 10.1186/s13321-020-0416-x (PMC7015965; doi:10.1186/s13321-020-0416-x)
Supplement: Supplementary file 1 — Additional file 1. Additional figures. [file 13321_2020_416_MOESM1_ESM.docx]

**Supplementary Information**


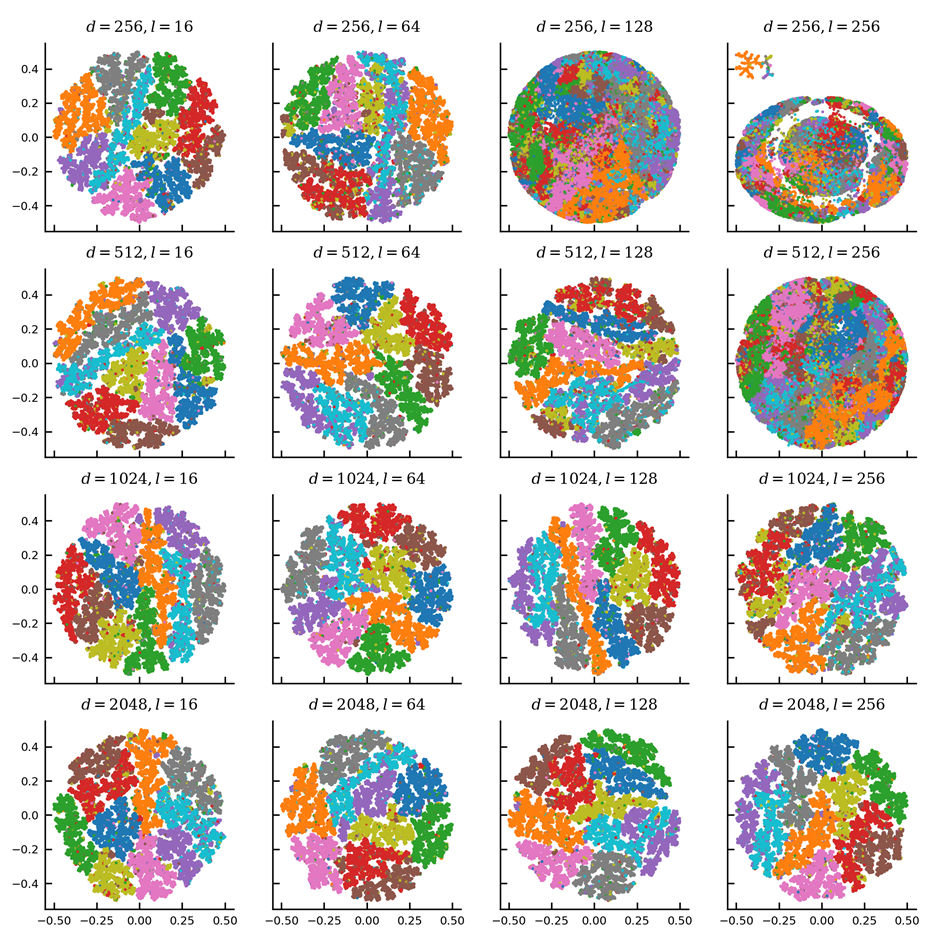


**Fig. S1 Influence of LSH Forest parameters** $\boldsymbol{d}$ **and** $\boldsymbol{l}$ **on visualization of MNIST**. While phase I of the algorithm mainly influences the preservation of locality (Fig. S6), extreme values where $d\approx l$ lead to a deterioration of visualization quality.


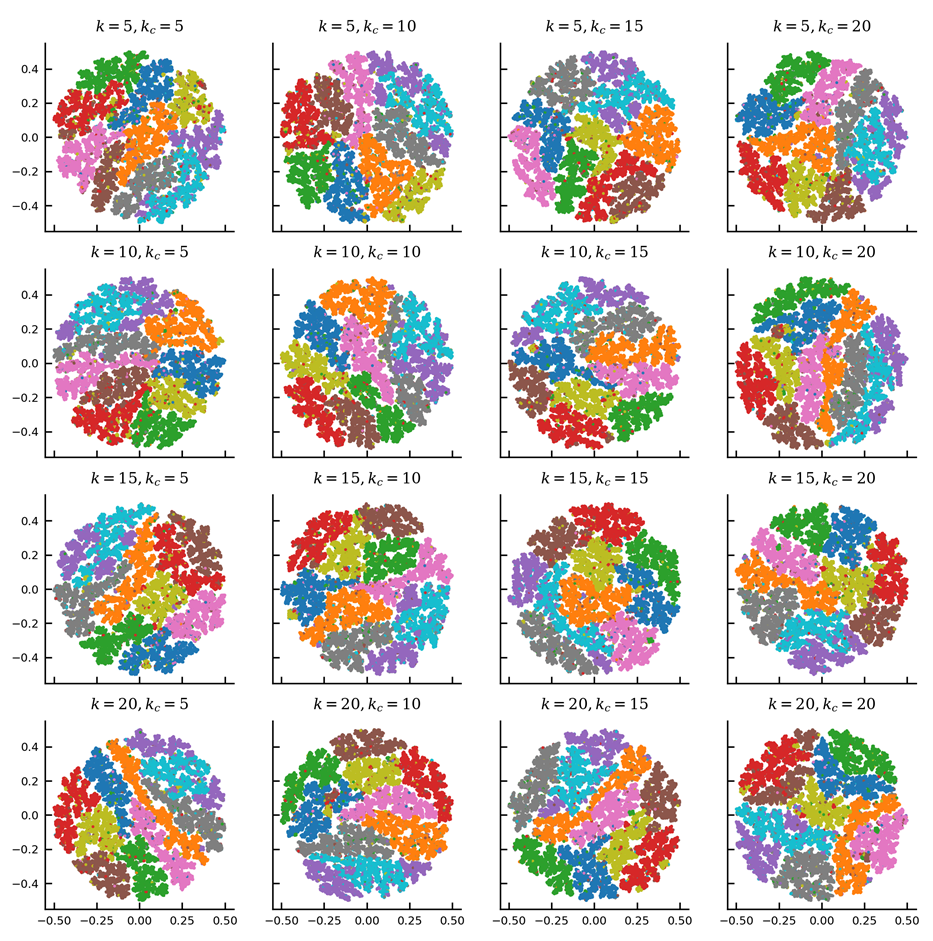


**Fig. S2 Influence of LSH Forest parameters** $\boldsymbol{k}$ **and** $\boldsymbol{k}_{\boldsymbol{c}}$ **on visualization of MNIST**. Whereas parameter $k$ directly influences the average degree of the $k$-nearest neighbor graph, $k_{c}$ increases the quality of the returned $k$ nearest neighbors. Both parameters only marginally influence the aesthetics and quality of the visualization.


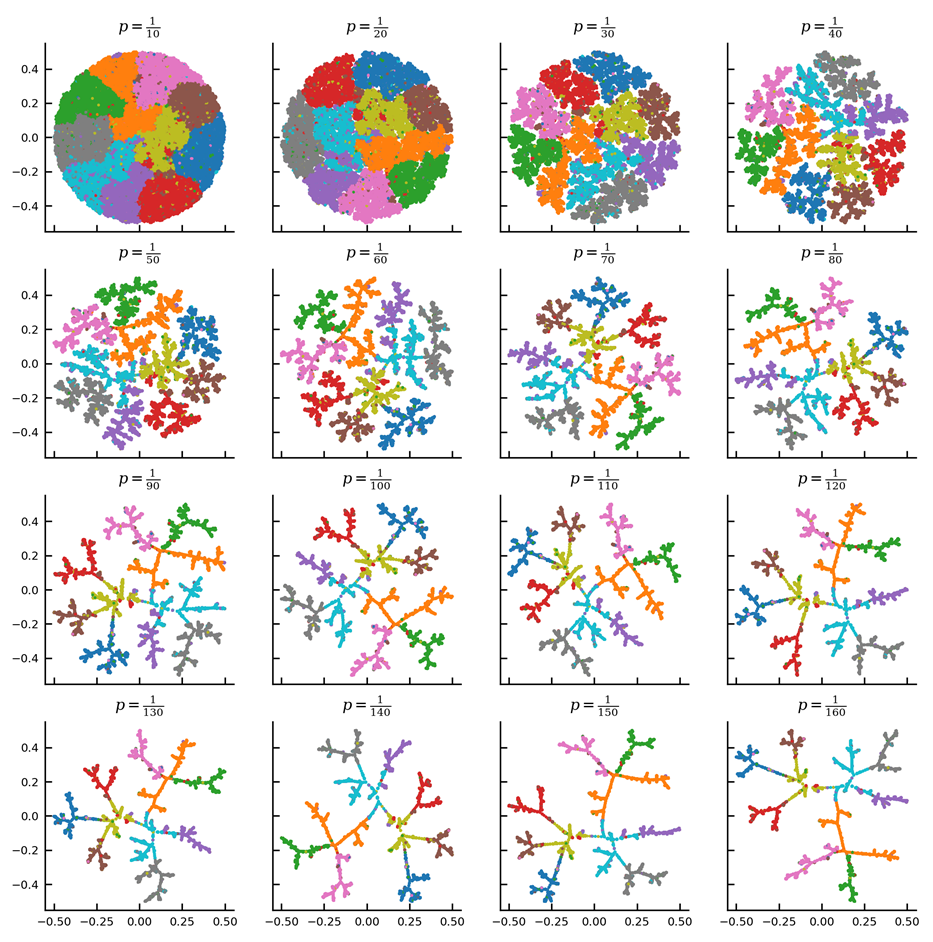


**Fig. S3 Influence of parameter** $\boldsymbol{p}$ **on visualization of MNIST**. The point size parameter $p$ has major influence on the aesthetics of the visualization, as it controls the sparseness of the drawn tree. Decreasing the point size and thus the repulsive force between two points, allows the layout algorithm to draw points closer to their respective (sub) branches.


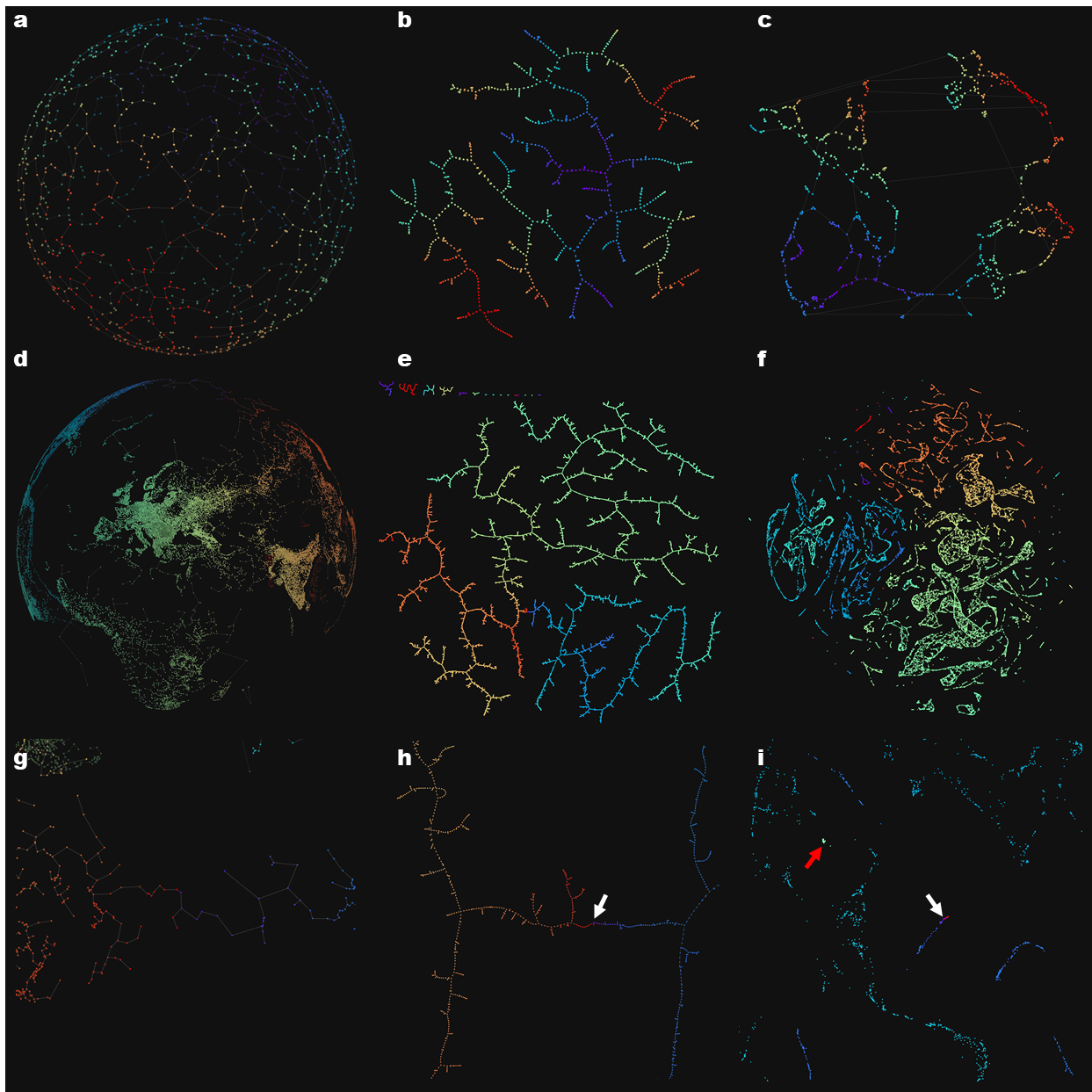


**Fig. S4 Examples of 2D embeddings of points sampled from the surface of 2-spheres by TMAP and UMAP**. (a, b, c) Colored by the points x-components in the original 3-dimensional Euclidean space, (d, e, f) colored by longitude. (a) Randomly distributed points picked from the surface of a 2-sphere were embedded on the two-dimensional plane by TMAP (b) and UMAP (c) using an angular metric in 0.859s and 2.96s wall-clock time respectively. Both algorithms were run with their respective nearest neighbor parameters set to 10. The results clarify the intrinsic need to break locality when embedding unbounded closed surfaces. (d) The location of cities with a population higher than 1,000 ($n=47980$) mapped to a sphere. (e) TMAP and (f) UMAP embeddings of the city-representing points in a 2D plane. The colors roughly represent Europe and Africa (green), India and central Asia (yellow), east Asia (orange), Oceania (red), and the Americas (blue). Using a Euclidean metric, the execution time of the algorithms was 18.566s and 218.528s, respectively. A detailed view of the Bering Strait (white arrows) in original 3D space with TMAP edges added (g) embedded by TMAP (h) and UMAP (i) highlights the differences between the two approaches. As laying out a graph is vastly more complex than laying out a tree, UMAP produces embedding errors such as the placement of Sardinian cities surrounded by North American cities (red arrow).


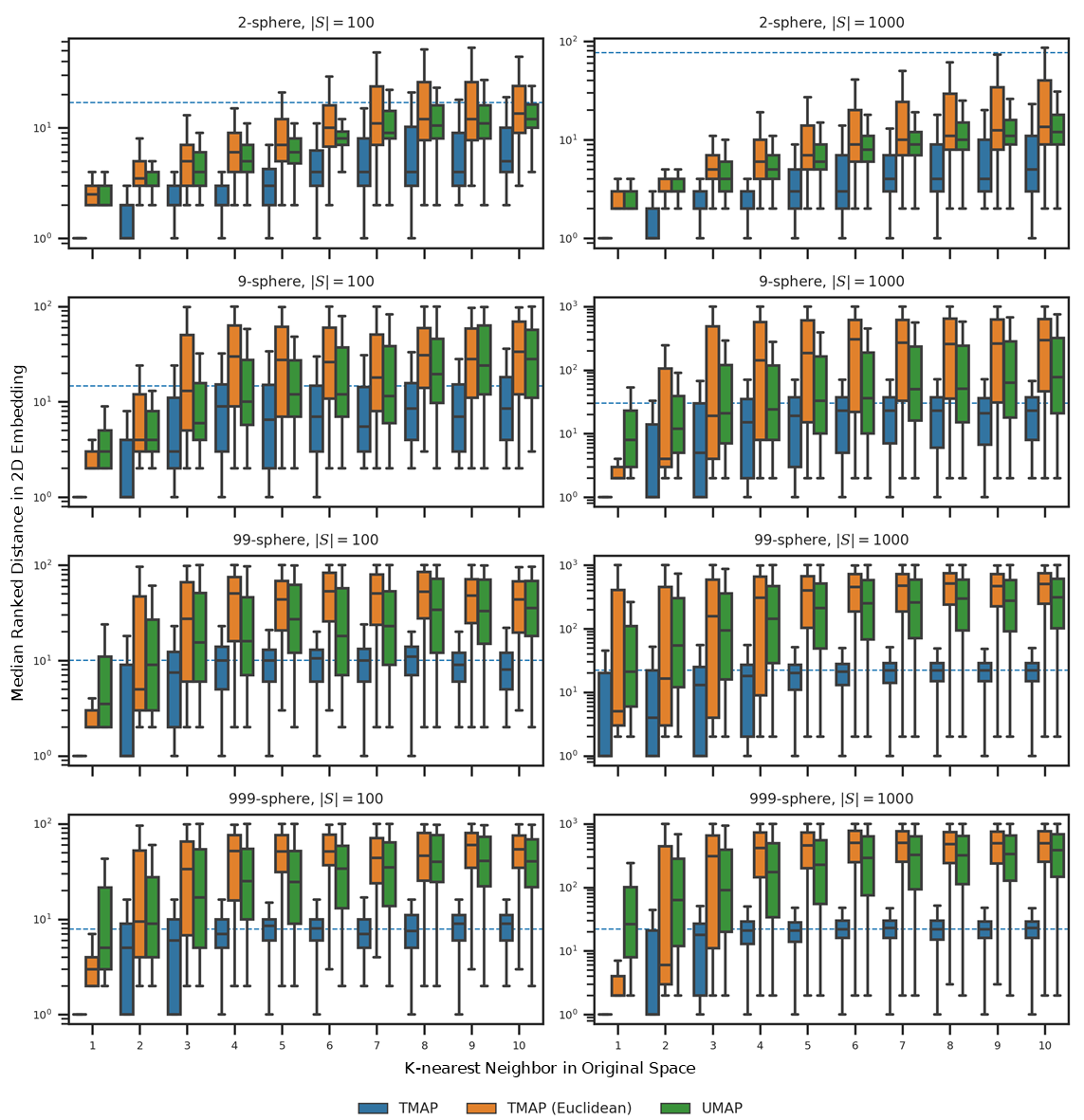


**Fig. S5 Locality-preservation in 2D embeddings of** $\boldsymbol{n}$**-spheres by TMAP and UMAP**. Random points were uniformly picked from the surface of $(n-1)$-spheres with $n\in\left\{ 3, 10, 100, 1000 \right\}$. In addition, the number of points were varied between 100 and 1,000. These examples represent edge cases due to the nature of high dimensional hyperspheres. For each point, the 10 nearest neighbors in the original $n$-dimensional space were compared to their ranked distance in the 2-dimensional plane. As the topological distance cannot be directly compared to the two Euclidean distances given the very high likeliness of collisions at any given (topological) distance due to branching, the average topological distance between any two points was included (blue dashed line).The quality in locality preservation of both TMAP and UMAP degenerates quickly in higher dimensions, however, TMAP tends to preserve the two nearest neighbors even when embedding very high dimensional data.


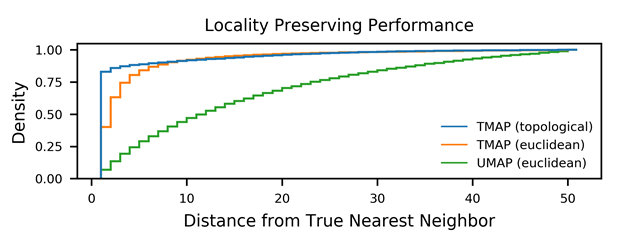


**Fig. S6 Ranked distance from true nearest neighbor when visualizing the MNIST data set**. Ranked distances from true nearest neighbor in original high dimensional space after projection based on topological and Euclidean distance for the MNIST data set. Whereas UMAP preserves less than 10% of true 1-nearest neighbors, TMAP preserves more than 80% based on topological and more than 35% based on Euclidean distance.


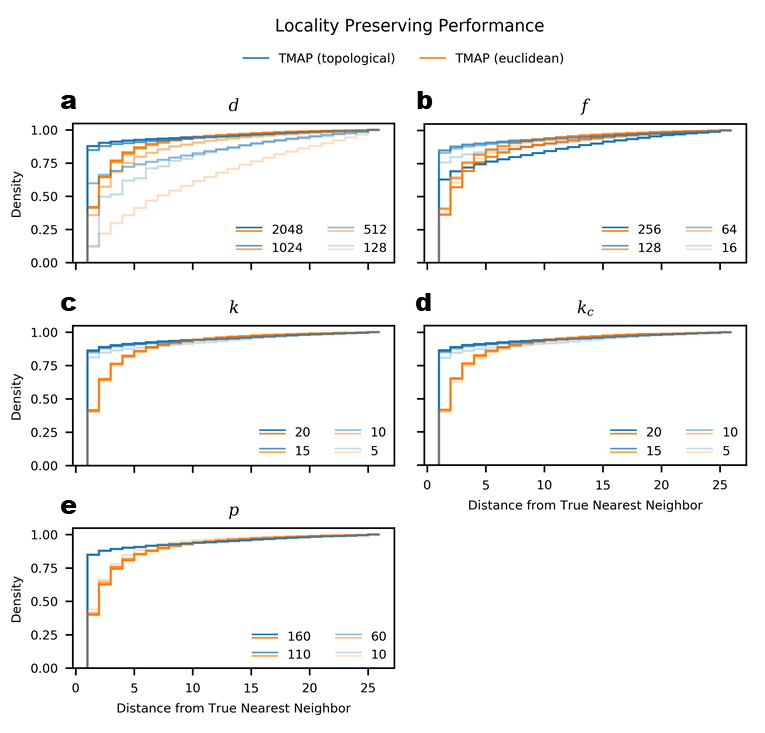


**Fig. S7 Influence of TMAP parameters on locality preserving performance**. Ranked distances from true nearest neighbor in original high dimensional space after projection based on topological and Euclidean distance for the MNIST data set. While, parameters $d$ and $l$ (**a**, **b**) have a major influence on both, the topological and Euclidean measure of locality preserving performance, parameters $k$ and $k_{c}$ have only marginal influence (**c**, **d**). The point size $p$ does not influence topological distances; however, it has a minor effect on the Euclidean distance-based metric, as higher values increase the sparsity of the drawn tree.


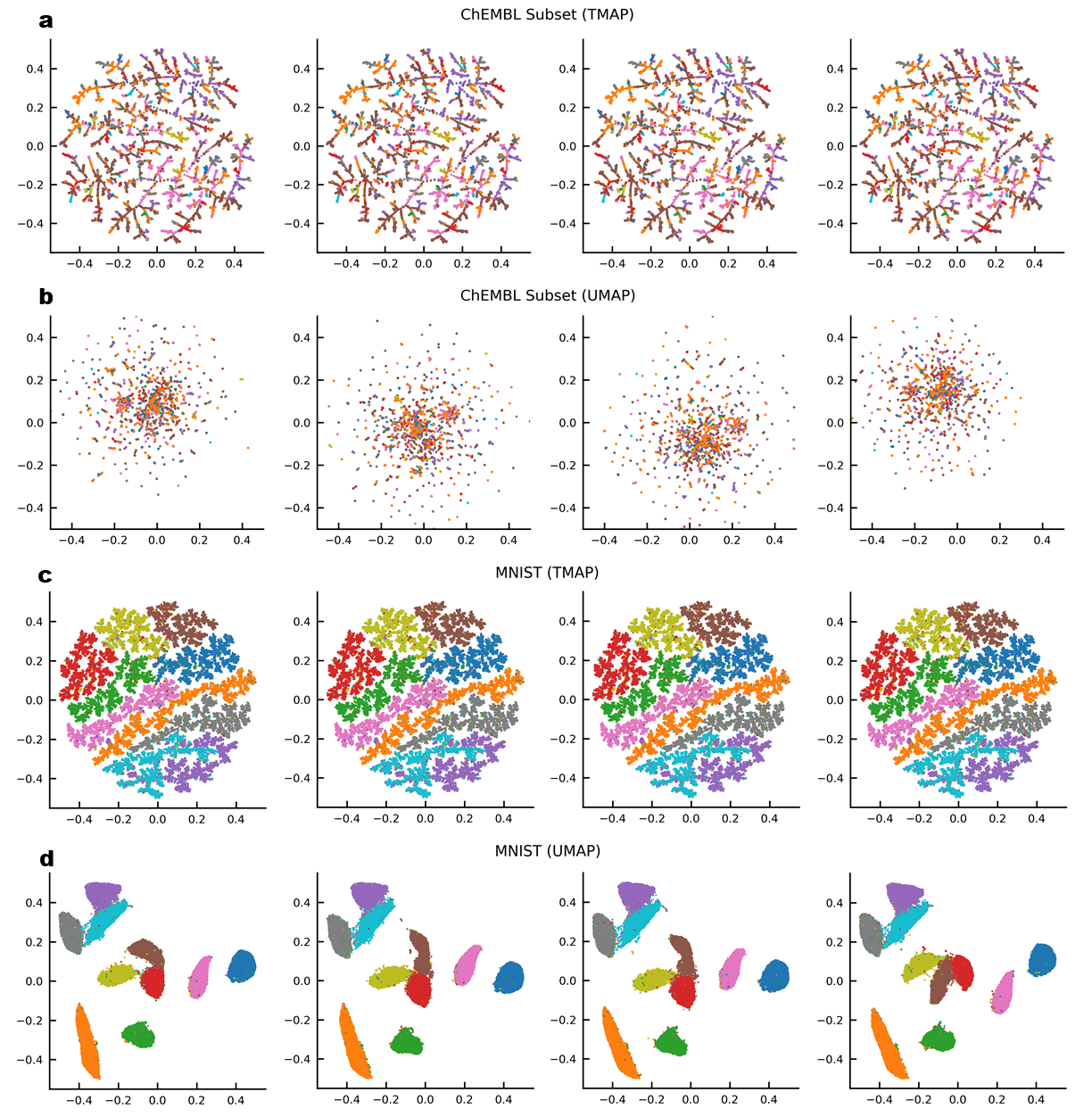


**Fig. S8 Stability of TMAP**. Algorithms TMAP (**a**, **c**) and UMAP (**b**, **d**) have been repeatedly ($n=4$) run on the same data sets with the same parameters. Whereas the output of TMAP is perceived as identical in all instances, the results yielded by UMAP show considerable differences between each run.
